# Cucurbitane-Type Glycosides and Sterol from *Momordica balsamina* Linn. As Target Potential Leads for Diabetes Management

**DOI:** 10.3390/molecules31081231

**Published:** 2026-04-08

**Authors:** Buang Matseke, Daniel Tswaledi, Raymond T. Makola, Xavier Siwe-Noundou, Ali H. Rabbad, Sekelwa Cosa, Kokoette Bassey

**Affiliations:** 1 Department of Pharmaceutical Sciences, School of Pharmacy, Sefako Makgatho Health Sciences University, Ga-Rankuwa, Pretoria 0204, South Africa; xavier.siwenoundou@smu.ac.za (X.S.-N.); kenubjeff@yahoo.com (K.B.); 2 Department of Biochemistry and Biotechnology, School of Science and Technology, Sefako Makgatho Health Sciences University, Ga-Rankuwa, Pretoria 0204, South Africa; 201012957@swave.smu.ac.za; 3 Department of Biochemistry, Microbiology and Biotechnology, University of Limpopo, Sovenga, Polokwane 0727, South Africa; raymond.makola@ul.ac.za; 4 Department of Biochemistry, Genetics and Microbiology, University of Pretoria, Hatfield, Pretoria 0028, South Africa; alihassan.rabbad@up.ac.za (A.H.R.); sekelwa.cosa@up.ac.za (S.C.)

**Keywords:** diabetes mellitus, *Momordica balsamina*, compound isolation, α-amylase, α-glucosidase, β-glucosidase, molecular docking, cytotoxicity

## Abstract

*Momordica balsamina* Linn. is widely used in traditional medicine for the management of diabetes; however, the specific bioactive compounds responsible for this activity have not been fully isolated and structurally elucidated from South African populations. This study reports, for the first time, the isolation and comprehensive characterization of antidiabetic compounds from South African samples of *M. balsamina*. Crude extracts were obtained through sequential solvent extraction, followed by isolation and purification using vacuum liquid chromatography. Structural elucidation was achieved using HPLC, UPLC–MS, FTIR, and NMR spectroscopy. The antidiabetic potential of the isolated compounds was evaluated through inhibition assays against α-amylase, α-glucosidase, and β-glucosidase. Molecular docking was employed to examine binding interactions with these target enzymes, while cytotoxicity was assessed using the MTT assay against Vero and HEK-293 cell lines. Two compounds, DD26.27 and EAEA1.2, were successfully isolated from dichloromethane and ethyl acetate extracts, respectively. Both compounds demonstrated concentration-dependent inhibition of the tested enzymes. Notably, molecular docking revealed strong binding affinities and favorable interactions with key catalytic residues, surpassing the standard drug acarbose and corroborating the in vitro results. Cytotoxicity studies confirmed that, at lower concentrations, the compounds were non-toxic to the tested cell lines. Collectively, these findings provide novel scientific validation of the traditional use of *M. balsamina* in South Africa and identify promising lead compounds for further in vivo studies and antidiabetic drug development targeting postprandial hyperglycemia.

## 1. Introduction

The current conventional drugs used for the treatment and management of diabetes are associated with significant hurdles, including high costs for low-income individuals and problems associated with prolonged use leading to adverse effects, reduced efficacy, poor patient compliance and safety concerns [1,2]. These drugs target postprandial hyperglycemia (PPHG), a state in which the blood glucose levels remain high after consuming a meal [3]. PPHG plays a role in the onset of type 2 diabetes (T2DM), and it is also associated with secondary diabetes complications such as neuropathy, retinopathy, cardiovascular diseases, etc. [4,5]. Carbohydrate-digesting enzymes such as α-amylase and α-glucosidase as well as β-glucosidase play a role in the breakdown of complex sugars into absorbable sugars resulting in PPHG. Inhibitors of these enzymes, such as acarbose, miglitol and voglibose, have been used in the management of PPHG; however, they have been reported to possess gastrointestinal side effects [6]. With the severe side effects reported for these conventional medications, the search for affordable alternative inhibitors with improved safety profiles, such as those derived from medicinal plants, has intensified.

*Momordica balsamina* Linn (*M. balsamina*), commonly known as balsam apple, is a wild climber widely cultivated for its biological and nutritional values [7,8]. *M. balsamina* is used traditionally across African countries for the treatment and management of diabetes, high blood pressure and other related ailments [9]. The leaf extracts have been reported to possess several biological activities including but not limited to antioxidant, antiviral, anti-inflammatory and antidiabetic activity [10,11,12]. The phytochemical profiles of species from other African regions have revealed the presence of cucurbitane-type triterpenoids, glycosides, sterols, flavonoids, and other secondary metabolites with reported antidiabetic, antioxidant, and anti-inflammatory activities. Among these, cucurbitane-type glycosides are particularly noteworthy due to their structural diversity and documented biological activities, including enzyme inhibition and modulation of glucose metabolism [7,8].

Molecular docking is a computer-based technique used in predicting the interactions between a small molecule (ligand) and the target macromolecule (protein/enzyme) at their atomic levels [13,14]. In this study, molecular docking predicts the binding mode and affinity of the isolated compounds within the active sites of the target enzymes α-amylase, α-glucosidase and β-glucosidase, thus providing the insight into inhibitory activity of isolated compounds at the molecular level [15,16]. When molecular docking is interpreted together with the experimental data and cytotoxicity evaluation, the results simultaneously assess the preliminary safety, efficacy and target specificity, which is essential for prioritizing the isolated compounds for further development [17,18].

Despite all the phytochemicals reported and its effective traditional use, there is still limited scientific evidence linking specific isolated compounds from *M. balsamina* to defined mechanisms of antidiabetic action. The current study therefore focuses on isolating and characterizing bioactive compounds from *M. balsamina* leaf extracts and assessing their antidiabetic potential through α-amylase, α-glucosidase and β-glucosidase inhibitory assays, performing molecular docking with these specific enzymes and further evaluating their safety on Vero and Hek-293 cells through an MTT assay.

## 2. Results

### 2.1. Fractioning, Isolation and Structural Characterization of Compounds DD26.27 and EAEA1.2

**Figure 1:**
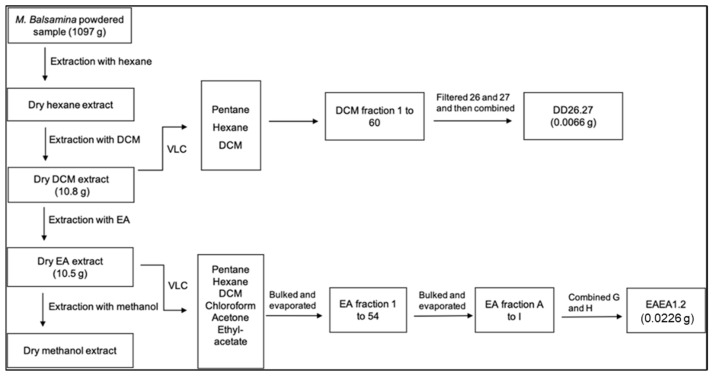
Flow chart representing isolation of compounds DD26.27 and EAEA1.2 from DCM and EA leaf extracts.

The dry plant powder, 1097 g, was VLC fractionated using hexane, dichloromethane (DCM), ethyl acetate (EA) and MeOH. The masses of the resulting dry enriched fractions are shown in Figure 1. DD26.27 and EAEA1.2 were isolated from enriched DCM and EA extracts respectively.

#### 2.1.1. Fractionation and Isolation of Compound DD26.27

Compound DD26.27 was isolated using open column chromatography by collecting test tube fractions. From a total of 34 test tube fractions, in the TLC analysis, test tubes 26 and 27 were bulked together because both afforded a single compact spot with an Rf value of 0.6 from DCM used as the mobile phase.

##### Fourier Transform Infrared Spectroscopy

Fourier transform infrared spectroscopy (FTIR) spectral analysis was carried out to determine the functional groups present in compound DD26.27. The detection of the functional groups was based on the peak values in the region of infrared radiation. The FTIR spectrum of compound DD26.27 demonstrated nine major peaks, at 3348.13 cm^−1^, 2957.01 cm^−1^, 2923.83 cm^−1^, 2852.12 cm^−1^, 1726.76 cm^−1^, 1464.24 cm^−1^, 1187.49 cm^−1^, 1077.86 cm^−1^ and 968.23 cm^−1^. The spectrum showed a weak broad peak at 3348.13 cm^−1^, indicating the presence of a hydroxyl group (O-H). There were three peaks, at 2957.01 cm^−1^, 2923.83 cm^−1^ and 2852.12 cm^−1^, displayed within the frequency range of 3000–2850 cm^−1^ corresponding to C-H stretching vibrations of -CH3 and -CH2- groups of a hydrocarbon backbone. The observed peak at 1726.76 cm^−1^ may represent a C=O group; however, since there is no other carbonyl-related absorption, this might be representing either a combination peak or trace impurities. The peaks at 1464.24 cm^−1^, 1187.49 cm^−1^ and 1077.86 cm^−1^ within the range of 1500 to 1000 cm^−1^ indicated C-O stretching and thus confirmed the presence of oxygenated functional groups such as alcohol, ester or ether. The last peak at 968.23 cm^−1^ may be attributed to an alkene or aromatic functional group in the compound. These results suggest that compound DD26.27 contains hydroxyl, carbonyl, and possibly aromatic or unsaturated components in its chemical structure.

##### High-Pressure Liquid-Chromatography of Compound DD26.27

To confirm the purity of DD26.27, an HPLC analysis was conducted. The HPLC chromatogram of DD26.27 revealed a major single peak that resolved at a retention time (Rt) of 4.2 min. There are minor peaks observed at Rt of 1.5 and 1.6 min that can be attributed to impurities, experimental conditions or column conditions. These factors do not affect the purity of compound DD26.27 as the percentage purity calculated was 97% purity, which is considered good for a compound that has been isolated from a natural product [EB108.1].

##### Ultra-Performance Liquid Chromatography–Mass Spectrometry (UPLC-MS)

Ultra-performance liquid chromatography–mass spectrometry (UPLC-MS) [EB110.1] was used to determine the estimated molecular formula, molecular mass and possible fragmentation patterns of compound DD26.27. The high-resolution electrospray ionization time-of-flight mass spectrometry (HR-ESI-TOF-MS) spectrum displayed in a negative ion mode exhibited a major deprotonated molecular ion with a mass-to-charge ratio (*m*/*z*) of 326.1898 ([M − H]^−^), suggesting that the compound might be an oxygenated organic compound. The presence of one or more carbon–carbon double bond (=) supported by the degree of unsaturation is in agreement with the alkenes detected by FTIR. There are fragmented ions with mass-to-charge ratios of *m*/*z* 311.1713 and 283.2730, demonstrating that there is a possible alkyl chain cleavage of an unsaturated, oxygenated phytochemical, thus supporting that the molecular formula is correct.

#### 2.1.2. Structural Elucidation of DD.26.27

DD26.27 was isolated as a white solid, UVCHCl3 λmax 226, 288 nm. The one-dimension ^1^H NMR (600 MHz, Methanol-d4), ^13^C NMR (150 MHz, Methanol-d4) and DEPT, as well as the two-dimensional COSY, HSQC and HMBC, of DD26.27 were interpreted with the help of a Mestre Nova^®^ and ACD labs structure elucidator. The chemical shifts, multiplicity and coupling constant of DD26.27 are listed in Table 1. HRESIMS (negative ion mode) *m*/*z* 325.2698 [M + H] (Calcd for C_23_H_32_O 325.5297) was recorded for DD26.27. HRESIMS fragmentation pattern *m*/*z*: 325 (M + 1), 311 (M—C_22_H_31_O), 283 (M—C_20_H_27_O), 180 (M—C_13_H_24_), 146 (M—C_10_H_10_O). An Rf value of 0.23 was obtained for DD26.27 by TLC using Hex: EA: ACTN (6:2:2 *v*/*v*/*v*) as the mobile phase.

##### Proton NMR of DD26.27

The number of protons (H) in DD26.27 integrated to 34. Amongst these protons were three phenyl protons that signal at δ 7.12 (H, d, *J* = 6 Hz), 7.40 (H, d, *J* = 6 Hz), and 7.58 (H, s) ppm. In addition to these phenyl protons were mostly saturated protons that signaled between 0.83 ppm and 2.39 ppm. This set of protons was presumed to comprise methyl, methylene and methyl protons considering their chemical shifts. In particular, there were characteristic double methyl terminal triplet protons that signaled at δ 0.85 ppm (3 H, m) and at δ 1.2 ppm (3 H, m).

##### Carbon-13 NMR of DD26.27

The C-13 experiment of DD, just like in the Proton NMR experiments, displayed a single O-substituted carbon at 147.6 and five other phenyl carbons at 138.5, 138.5, 124.4, 123.9 and 119.1 ppm. Whereas the two carbons at 138.5 ppm were typical of bridging carbons, the others were a phenyl ring system of angular carbons. All the other carbons corresponded to the saturated system that appeared at 37.1, 34.8, 34.5, 32.7, 31.9, 31.4, 30.2, 30.0, 29.6, 29.6, 29.6, 29.3, 27.0, 26.7, 22.6, 19.7 and 14.0 ppm.

##### Distortedness Enhancement by Polarization Transfer (DEPT)

The one-dimensional DEPT experiment with DD26.27 was instrumental in identifying the number of CH_3_, CH_2_, and CH present in the DD26.27 compound. From the experiment, the numbers of CH_3_, CH_2_ and CH were elucidated as 2, 9 and 6, respectively. The DEPT experiments also revealed three aromatic SP^2^ phenyl CH atoms at δ 7.58, 7.40 and 7.12. The three carbon atoms that did not signal in the DEPT experiments were thought to be the phenyl CH atoms. To confirm these phenyl protons in DD26.27, the two-dimensional heteronuclear single quantum correlation (HSQC) NMR experiment was conducted to reveal carbons directly correlated with protons via one J coupling constant.

##### HSQC NMR of DD26.27

To investigate the presence of protonated and quaternary carbons that are present in the structure of DD26.27, HSQC experiments of DD26.27 were conducted. The results obtained suggested that DD26,27 comprises only three quaternary carbons at C3, C5 and C10 with d147.6, 138.5 and 138.5 for two quaternary carbons that were in the same chemical environment. Having established the number of protons, carbons and carbons attached on protons, the multiplicity and coupling constantans of these moieties DD26.27 are summarized in Table 1.

##### HMBC NMR of DD26.27

**Figure 2:**
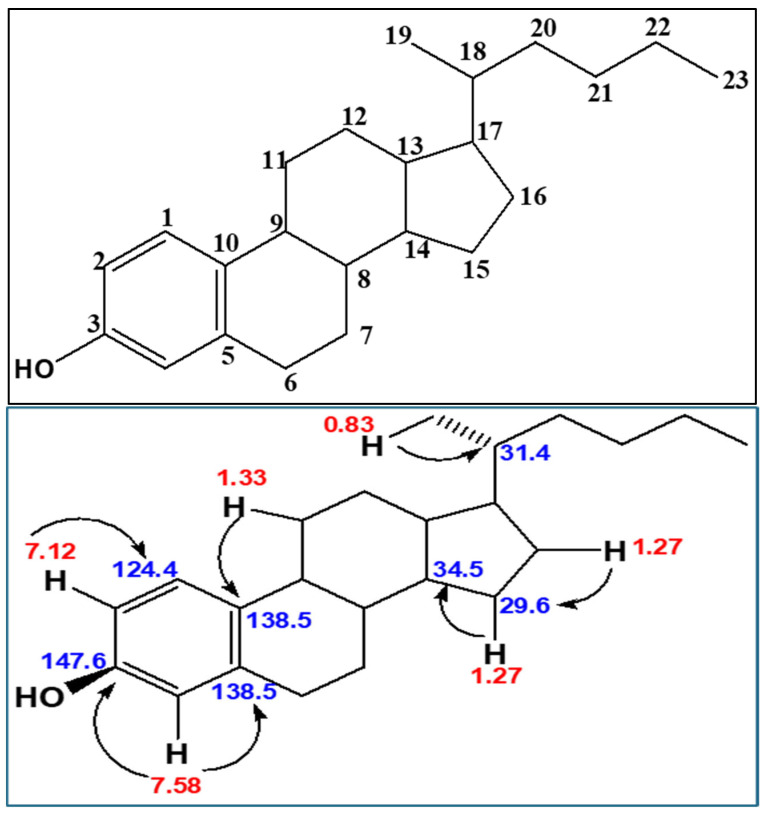
Carbon skeleton (**top**) and HMBC correlations (**bottom**) of compound DD26.27. (Red numbers represent hydrogens and blue numbers represent carbons).

This experiment is indispensable when it comes to knitting up the different moieties that make up the structure of an unknown molecule. At this juncture, the experiments were indicative of three moieties, i.e., a phenyl ring, a tricyclic and a saturated hexane system as part of the structure of DD26.27. The phenyl ring system correlated to the tricyclic moiety via 2*J* coupling of the phenyl H-4 proton at d 7.59 and the two-unit bridge carbon at d 138.5 ppm. The second crucial HMBC correlations confirm the linkage between the phenyl/tricyclic unit and the saturated hexane unit H-19 coupling with C-18. The other HBMC correlation that completes the connection between the three units that make up the structure of DD26.27 is displayed in Figure 2.

#### 2.1.3. Fractionation and Isolation of Compound EAEA1.2

Isolation of compound EAEA1.2 was achieved using open column chromatography by collecting fractions in the test tubes. About 54 fractions were collected from the column, and TLC was performed for the collected fractions. The fractions were grouped together and marked fractions A to I. Fractions G and H afforded a single compact band with an Rf value of 0.4 and were then combined and named EAEA1.2. The combined fraction EAEA1.2 was run on 2D TLC, and it confirmed that it is one compound represented by a single band.

##### Fourier Transform Infrared Spectroscopy (FTIR)

The functional groups present in the structure of compound EAEA1.2 were determined by FTIR spectral analysis, which depends on the values of the peaks detected in the infrared region. The FTIR spectrum of compound EAEA1.2 exhibited eight major peaks, at 3305.47 cm^−1^, 2933.31 cm^−1^, 2856.86 cm^−1^, 1693.58 cm^−1^, 1645.58 cm^−1^, 1373.57 cm^−1^, 1263.94 cm^−1^ and 1226.01 cm^−1^. The sharp peak observed at 3305.47 falls within the frequency ranges of 3650–3250 cm^−1^, which represent the free O-H stretching vibrations or possible N-H stretching vibrations. Absorption peaks at 2933.31 cm^−1^ and 2856.86 cm^−1^ may be attributed to alkanes’ functional group (C-H), confirming the presence of aliphatic moieties in the chemical structure. A peak at 1693.58 cm^−1^ was observed, falling within the frequency range of 1725–1650, thus indicating the presence of C=O, and suggesting the presence of carbonyl functional groups such as ketones, amide or esters. Within a frequency range of 1680–1620 cm^−1^, there is one peak at 1645.58 cm^−1^, which can be attributed to C=C stretching vibrations of alkenes, indicating the presence of unsaturated carbon–carbon double bonds. The three peaks at 1373.57 cm^−1^, 1263.94 cm^−1^ and 1226.01 cm^−1^ indicate C-O stretching, suggesting the presence of alcohols, ethers, carboxylic acid or esters. Overall, the results indicate that compound EAEA1.2 contains hydroxyl, aliphatic, carbonyl, and ether functional groups.

##### High-Pressure Liquid Chromatography (HPLC)

The obtained HPLC chromatogram for EAEA1.2 showed a major single peak resolved at Rt 13.5 min. A minor peak was observed at Rt 0.15 min with some background noise between 2.5 and 7.5 min. The purity percentage was determined at 97%, which is highly acceptable for a compound isolated from natural products. Because the purity of the major peak was found to be 97%, the observed minor peak and background noise were attributed to experimental conditions or rather column conditions.

##### Ultra-Performance Liquid Chromatography–Mass Spectrometry (UPLC-MS)

UPLC-MS was used to determine the estimated molecular formula, molecular mass and possible fragmentation patterns for compound EAEA1.2. The HR-ESI-TOF-MS spectrum recorded in a negative ion mode (ES+) demonstrated a dominant peak at a mass-to-charge ratio of *m*/*z* 663.4553. The calculated accurate mass and predicted molecular formula correspond with a compound of high molecular weight and high oxygenation.

#### 2.1.4. Structural Elucidation of EAEA1.2

Compound EAEA1.2 was isolated as a white solid, UVCHCl3 λmax 226, 288 nm. The one-dimensional ^1^H NMR (600 MHz, Methanol-d4), ^13^C NMR (150 MHz, Methanol-d4), and DEPT, as well as the two-dimensional COSY, HSQC and HMBC, of EAEA1.2 were interpreted with the help of a Mestre Nova^®^ and ACD labs structure elucidator. HRESIMS (negative ion mode) *m*/*z* 664.82 [M + H] (Calcd for C_36_H_56_O_11_) was recorded for EAEA1.2. HRESIMS fragmentation pattern *m*/*z*: 664 (M + 1), 623 (M—C_33_H_21_O_11_), 323 (M—C_24_H_35_), 341 (M—C_12_H_21_O_11_), 41 (M—C_3_H_5_). An Rf value of 0.45 was obtained for EAEA1.2 by TLC using chloroform: methanol (9:1 *v*/*v*) as the mobile phase.

##### Proton NMR of EAEA1.2

The number of protons (^1^H) in EAEA1.2 integrated to 56. The signals were indicative of a molecule consisting of saturated, sugar and olefinic moieties. In particular, the signals suggested the presence of two distinct anomeric protons at δ 4.42 (H, d, *J* = 12 Hz) and 5.15 (H, d, *J* = 14 Hz) ppm. The first anomeric proton displayed a JH1′-H-3 coupling constant, thus indicating that the tetracyclic moiety of EAEA1.2 was β-linked to the first sugar moiety. The (4′→1″)-coupling of the two sugar units confirms α/β-linkage via a coupling constant of *J* = 3 Hz. The presence of the two sugar units was further underscored by two geminal protons that signaled at δ 3.79, 3.54 (2H, *J* = 12 Hz) and 3.79; 3.54 (2H, *J* = 12 Hz) ppm is typical of hexoses. The olefinic protons of the molecule signaled at δ 5.29 (H, d, *J* = 12 Hz), 5.25 (H, dd, *J* = 12 Hz, 11.8 Hz), 6.67 (H, t, *J* = 12 Hz, 6 Hz), 6.39 (H, m), and 5.36 (H, m) ppm to suggest the presence of non-phenylic multiple bonds in the proposed structure of EAEA1.2.

##### Carbon-13 NMR of EAEA1.2

The C-13 experiment of EAEA1.2, just like the proton NMR experiments, displayed a total of 36 carbon signals. The signals were indicative of a molecule consisting of saturated, sugar and olefinic moieties. In particular, the signals suggested the presence of two distinct anomeric carbons at d 96.7 and 92.5 ppm. The presence of the two sugar units were further affirmed by two O-substituted carbons at d 61.5 and 61.5 ppm that are typical of hexoses. The olefinic units of the molecule signaled at d 114.6, 117.2, 118.0, 121.5, 129.4 and 129.4 ppm to suggest the presence of three double bounds in the proposed structure of EAEA1.2.

##### Distortedness Enhancement by Polarization Transfer (DEPT) of EAEA1.2

The one-dimensional DEPT experiment of EAEA1.2 was instrumental in indicating the number of CH3, CH2, and CH present in EAEA1.2. From the experiment, the numbers of CH3, CH2 and CH were elucidated as 3, 7 and 24 respectively. Whereas the CH3 signaled at 13.3, 22.3 and 25.7 ppm, the CH2 signals appeared at 29.0, 29.2, 29.3, 29.4, 29.6, 30.6 and 31.6 ppm, and those for the saturated CH methylene group pitched further downfield at 133; 22.3, 25.7, 30.6, 47.9, 48.0, 48.1, 48.3 and 48,.4 ppm to suggest that EAEA1.2 is characterized by a large, saturated moiety. In addition to the saturated carbons, there were four olefinic CH2 signals that resolved at 117.2, 121.5, 129.4 and 129.5 ppm. The carbons that did not appear in these experiments were presumed to be the quaternary carbons, two of which were in EAEA1.2, which are usually devoid of protons.

##### HSQC NMR of EAEA1.2

To investigate the presence of all one-J coupled protonated carbons and to confirm the number of quaternary carbons that are present in the structure of EAEA1.2, the HSQC experiment of EAEA1.2 was conducted. The results obtained confirmed that EAEA1.2 comprises only two quaternary carbons at C8 and C10 at d118.0 and 49.4 ppm, respectively. There were two sets of O-sugar protonated carbons at 96.7, 74.8, 72.4, 76.6, 71.5 and 92.5, 73.5, 70.5, 70.3, 76.6 ppm, respectively. The signals were peculiar to the presence of two sugar units in the structure of EAEA1.2 due to a pair of diagnostic C-6 sugar pairs of signals at d 61.5 ppm and their corresponding geminal protons.

##### HMBC NMR of EAEA1.2

**Figure 3:**
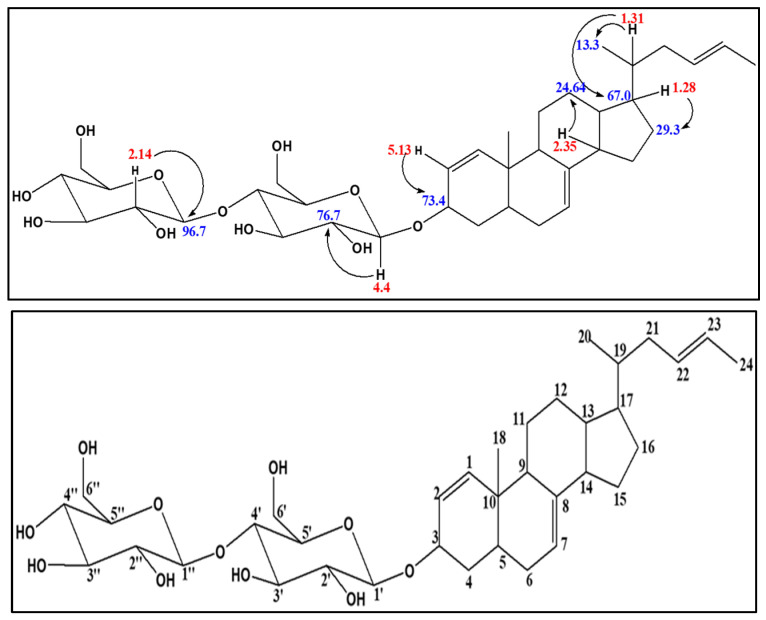
HMBC correlations (**top**) and the carbon skeleton (**bottom**) of compound EAEA1.2. (Red numbers represent hydrogens and blue numbers represent carbons).

This experiment is indispensable when it comes to knitting up the different moieties that makes up the structure of an unknown molecule. At this juncture, the experiments were indicative of three moieties, i.e., a tetracyclic steroidal skeleton, two sugar units and an unsaturated olefinic system as part of the structure of the molecule. These three moieties in EAEA1.2, using a maximum of the 3*J* coupling constant H-19 proton at 1.31 ppm connected to the C-13 at 67.0 ppm, as well as C-20 at 13.3 ppm, proved that the hexene unit in the EA.EA attached its steroidal tetracyclic unit. This combined units that connect to the sugar moiety via the steroid H-2 at 5.15 ppm, correlating with its C-3 at 73.4 ppm. The two sugar units linked to themselves through the connections of H-1′ at 4.4 and H-2″ at 3.14 to C-2′ at 74.8 and C-1″ at 92.5 ppm respectively. There were HMBC correlations between the protons and the carbons. When all the units of EAEA were knitted together, its skeletal structure was proposed, as evident in Figure 3, and its proton, carbon multiplicity and coupling constantans are summarized in Table 2 below.

**Figure 4:**
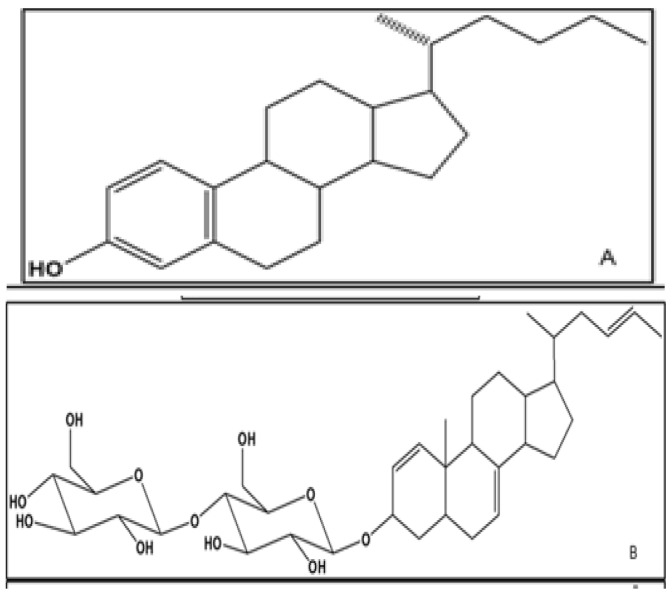
Structure of DD26.27 (17-(hexan-2-yl)-7,8,9,11,12,13,14,15,16,17-decahydro-6H-cyclopenta[a]phenanthren-3-ol) isolated from the DCM leaf extracts of *M. balsamina* (**A**) and structure of EAEA1.2 (-(6-(17-(E)-hex-4-en-2-yl)-4,5,6,9,10,11,12,13,14,15,16,17-dodecahydro-10-methyl-3H-cyclkuopenta[a]phenanthren-3-yloxy)-tetrahydro-4,5-dihydroxy-2-(hydroxymethyl)-2H-pyran-3-yloxy)-tetrahydro-6-(hydroxymethyl)-2H-pyran-3,4,5-triol) isolated from the ethyl acetate leaf extracts of *M. balsamina* (**B**).

Upon collating all the spectrometric and chromatographic data together, the proposed structure of DD26.27 was elucidated as 17-(hexan-2-yl)-7,8,9,11,12,13,14,15,16,17-decahydro-6H-cyclopenta[a]phenanthren-3-ol (Figure 4A). In like manner, EAEA1.2 was elucidated as 2-(6-(17-(E)-hex-4-en-2-yl)-4,5,6,9,10,11,12,13,14,15,16,17-dodecahydro-10-methyl-3H-cyclopenta[a]phenanthren-3-yloxy)-tetrahydro-4,5-dihydroxy-2-(hydroxymethyl)-2H-pyran-3-yloxy)-tetrahydro-6-(hydroxymethyl)-2H-pyran-3,4,5-triol (Figure 4B).

When the structures of DD26.27 and EAEA1.2 were compared, the similarities in their NMR data revealed that DD26.27 was possibly a bio glycosylated to afford EAEA1.2. The difference between the two compounds was majorly in the different positioning of the multiple bonds in their structures. Whereas the multiple bonds in DD26.27 alternated with single bonds to form a phenyl ring system, they were placed at C-1, C-4 and C-22 in EAEA1.2. A detailed and succinct literature search indicated that DD26.27 was an algycon of EAEA1.2. It was also clear that EAEA1.2 indicated similarities at R3 = glu-glu, steroid skeleton, hexane chain to cucurbit-5-en-3β-O-β-D-glucopyranosyl-(4′→1″)-O-β-D-glucopyranoside [19]. However, due to differences between both compounds in terms of the positioning of the olefinic and methyl groups, EAEA1.2 was elucidated as a sterol glycoside. Compound DD26.27 possesses a cyclopentanoperhydrophenanthrene nucleus characteristic of steroids and thus making it a steroidal alcohol (Sterol).

According to literature reports, compounds like cucurbit-5-en-3β-O-β-D-glucopyranosyl-(4′→1″)-O-β-D-glucopyranoside, which are cucurbitane glycosides from plants like *Siraitia grosvenori* (monk fruit) and other cucurbits, possess significant antidiabetic properties, helping to lower blood sugar, improve insulin sensitivity, and enhance glucose uptake, with effects comparable to some conventional medications. These natural compounds work by activating pathways like PI3K/AKT, promoting GLUT4 translocation for glucose absorption, and even stimulating insulin production, making them promising for diabetes management [19]. Factoring in on the properties of their analogues, the biological, and specifically the antidiabetic, potentials of DD26.27 and EAEA1.2 will be discussed in the next chapter.

### 2.2. Enzyme Inhibition Activity of Compound DD26.27

#### 2.2.1. Alpha-Amylase Inhibition Activity

**Figure 5:**
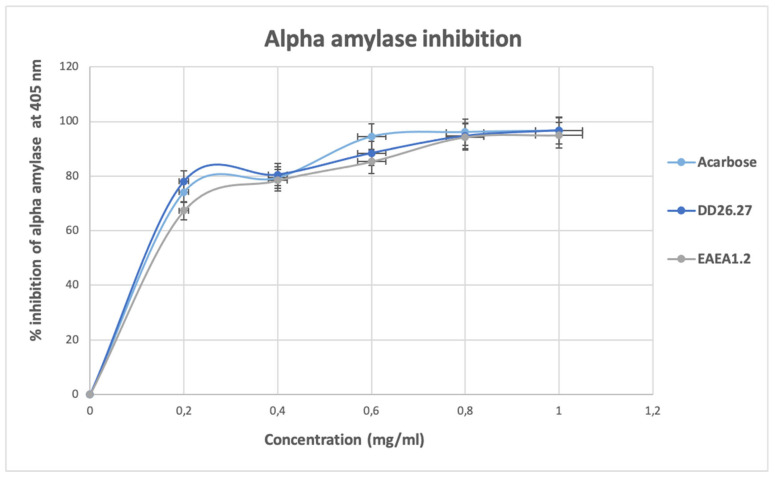
α-amylase inhibitory activity of standard drug acarbose and compounds DD26.27 and EAEA1.2.

Inhibition of α-amylase, as depicted in Figure 5, shows an increase in a concentration-dependent manner from the lowest concentration (0.2 mg/mL) up to a concentration of 0.8 mg/mL where they all reach a state of plateau. At this plateau state, with a concentration of 0.8 to 1.0 mg/mL, it is observed that the standard and two compounds DD26.27 and EAEA1.2 exhibited strong antidiabetic activity, reaching a near-maximum inhibition percentage above 95%. The results suggest that the active sites of α-amylase have been occupied and that a further increase in the compound will make no difference. At low concentrations tested, 0.2 to 0.4 mg/mL, compound DD26.27 (78.06% for 0.2 mg/mL and 80.5% for 0.4 mg/mL) outperformed the standard acarbose (74.18 for 0.2 mg/mL and 7.41 mg/mL), suggesting a potential natural alternative for controlling postprandial hyperglycemia. It is a good indication that the compound exhibits stronger antidiabetic potential at lower concentrations because excessive α-amylase inhibition has been associated with side effects such as gastrointestinal complications.

#### 2.2.2. Alpha-Glucosidase Inhibition Activity

**Figure 6:**
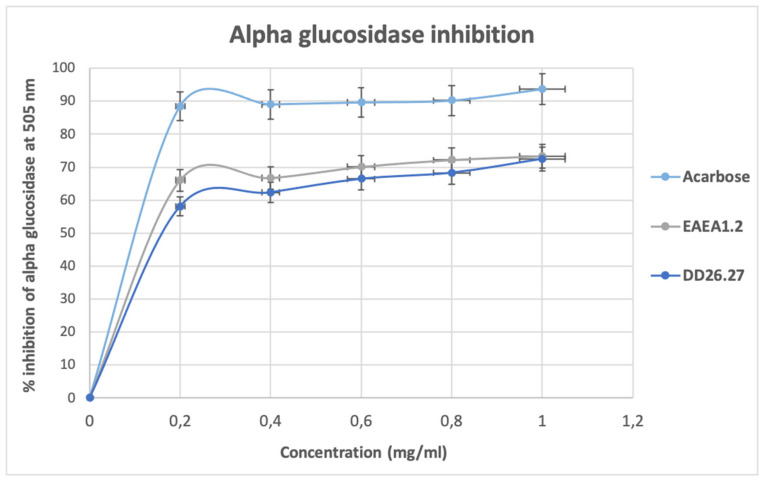
α-glucosidase inhibitory activity of standard drug acarbose and compounds DD26.27 and EAEA1.2.

The results of α-glucosidase inhibitory activity as depicted on Figure 6 show that the standard acarbose exhibited stronger inhibitory activity than the compounds DD26.27 and EAEA1.2 throughout all the concentrations tested (0.2–1.0 mg/mL). The α-glucosidase inhibitory activity of acarbose remains constant in all the concentrations, reaching over 85% at the lowest concentration of 0.2 mg/mL. The results suggest early enzyme saturation for acarbose, indicating a high binding affinity at very low concentrations and thus a low effective dose, minimizing the side effects. The standard acarbose was followed by compound EAEA1.2, which exhibited an α-glucosidase inhibitory activity of 66.04% to 73.36%, followed by compound DD26.27, which exhibited activity of 58.09% to 72.47% from the lowest concentration (0.2 mg/mL) to the highest concentration (1.0 mg/mL).

#### 2.2.3. Beta-Glucosidase Inhibition Assay

**Figure 7:**
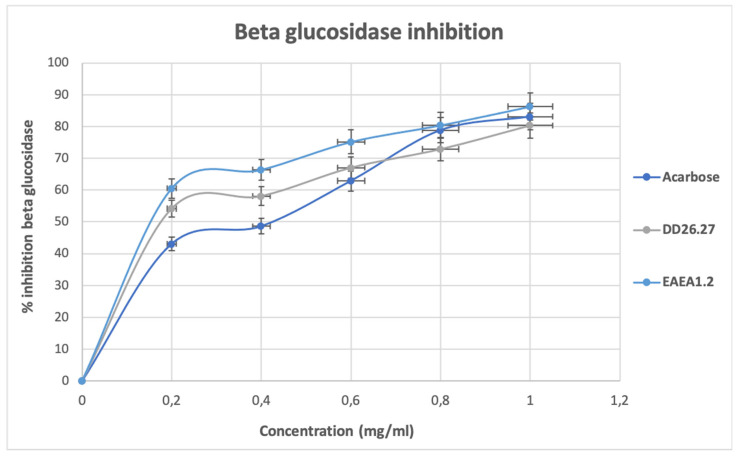
β-glucosidase inhibitory activity of standard drug acarbose and compounds DD26.27 and EAEA1.2.

The results of β-glucosidase, as depicted in Figure 7, shows an inhibitory activity for acarbose and compounds EAEA1.2 and DD26.27 show a concentration-dependent increase with compound EAEA1.2, exhibiting a higher percentage inhibition of β-glucosidase, and thus showing dominance from the lowest concentration (60.51%) to the highest concentration (86.27%) used in the test (0.2 to 1.0 mg/mL). Compound DD26.27 exhibited higher β-glucosidase inhibitory activity (54.18% to 67.04%) at concentrations of 0.2 to 0.6 mg/mL than acarbose, which exhibited inhibition percentages of 43.06% to 62.88% at the same concentrations. Overall, the compounds outperform the activity of the standard acarbose at low concentrations, indicating their potential as alternative natural postprandial hyperglycemia controls and that their effective inhibition at lower concentrations will minimize the side effects resulting from high dosages.

The IC_50_ values of compounds EAEA1.2 and DD26.27 were evaluated within the tested concentration range (0.2–1.0 mg/mL) and compared with that of the standard antidiabetic drug acarbose. For both α-amylase and α-glucosidase, all tested samples (EAEA1.2, DD26.27, and acarbose) exhibited inhibition greater than 50% at the lowest tested concentration (0.2 mg/mL). Consequently, the calculated IC_50_ values fall below 0.2 mg/mL and should be interpreted as indicative estimates rather than precise half-maximal inhibitory concentrations, as the lower portion of the dose–response curve was not captured. For β-glucosidase inhibition, compounds EAEA1.2 and DD26.27 similarly demonstrated IC_50_ values below the lowest tested concentration (0.2 mg/mL), indicating strong inhibitory activity under the tested conditions. In contrast, acarbose exhibited a measurable IC_50_ value of 0.424 mg/mL within the tested range.

Overall, although the IC_50_ values for several assays could not be precisely determined due to the limited lower concentration range, both EAEA1.2 and DD26.27 demonstrated potent inhibitory activity against the evaluated carbohydrate-hydrolyzing enzymes. These findings highlight their promising enzyme inhibitory potential, while acknowledging the methodological limitation associated with the current dose–response design.

### 2.3. Molecular Docking

The results from docking were analyzed to determine the binding affinities of the compounds with target enzymes, the amino acids interactions involved and the overall possible mechanism of action for potential enzyme inhibition. The results were compared with the standard inhibitor drug acarbose.

#### 2.3.1. Docking of Compounds with α-Amylase

**Figure 8:**
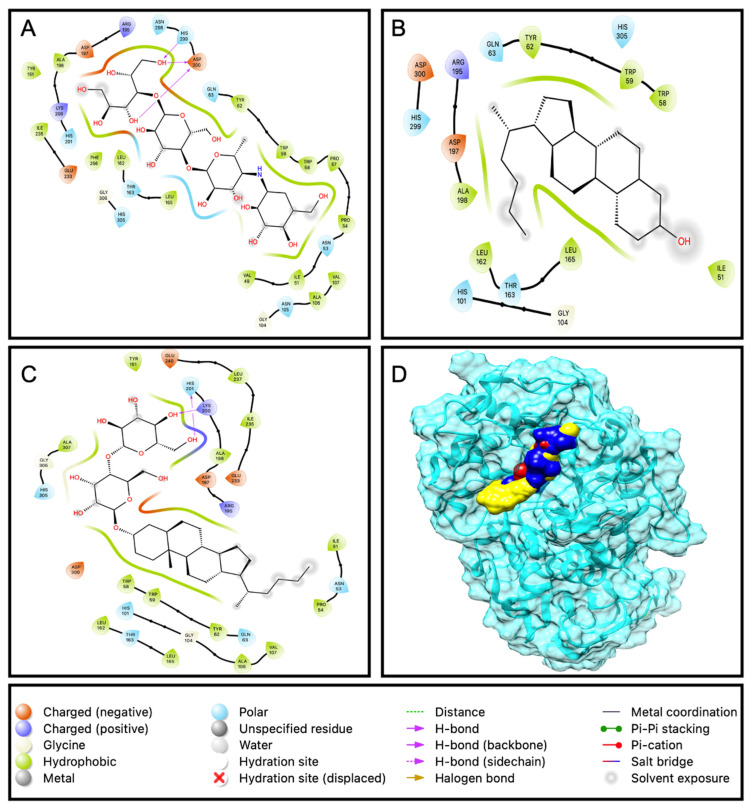
Ligand interaction with the amino acid residues of the active site for (**A**): acarbose, (**B**): DD36.37 and (**C**) EAEA1.2. Box (**D**) represents the protein α-amylase and all the ligands bound at the same pocket of the active binding site.

The docking results shows that all the compounds and the standard acarbose have the efficiency to bind with enzyme α-amylase. Compounds EAEA1.2 (−10.0 kcal/mol) and DD26.27 (−8.6 kcal/mol) exhibited better binding affinities than acarbose with the binding and −7.6 kcal/mol. This is in agreement with the results from an α-amylase inhibition assay that demonstrated compound EAEA1.2 showing stronger inhibition of the enzyme, with an IC_50_ of 0.201 μg/mL, than acarbose (0.209 μg/mL) and DD26.27 (0.25 μg/mL). Both the compounds and standard, as depicted in Figure 8. 2D, bind on the same pocket of the active site of the enzyme. Acarbose formed three hydrogen bonds (ASP 300 X2 and HIS299) within the active site, whereas EAEA1.2 formed two hydrogen bonds (LYS200 and HIS201). However, despite forming fewer hydrogen bonds, EAEA1.2 exhibited a superior binding affinity. This observation highlights that the hydrogen bond number alone does not determine the binding strength. Hydrophobic interactions, van der Waals forces, shape complementarity, and reduced electrostatic repulsion can contribute more significantly to the overall binding free energy than the mere count of hydrogen bonds.

#### 2.3.2. Docking of Compounds with α-Glucosidase

**Figure 9:**
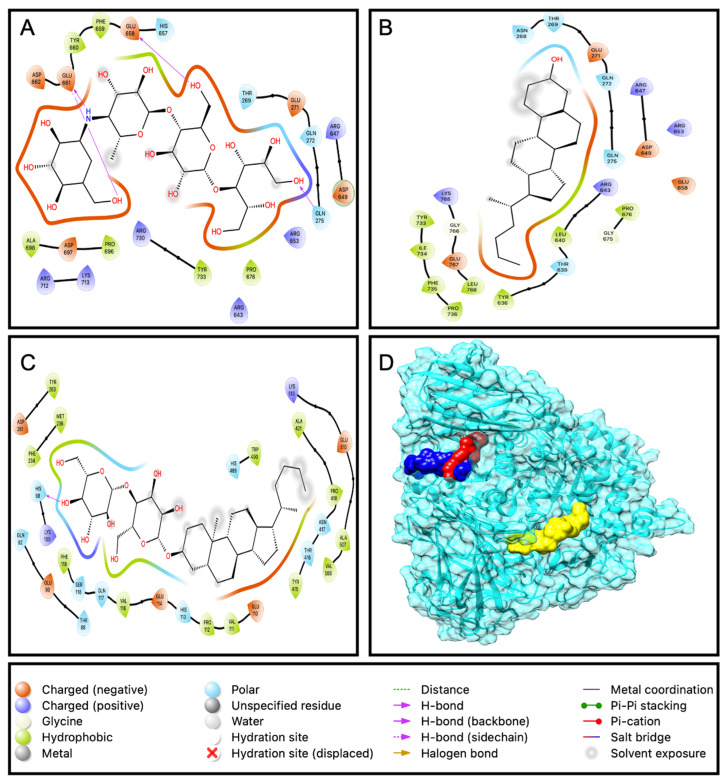
Ligand interaction with the amino acid residues of the active site for (**A**): acarbose, (**B**): DD36.37 and (**C**): EAEA1.2. Box (**D**) represents the protein β-glucosidase and all the ligands bound at the same pocket of the active binding site.

In the case of α-glucosidase, compounds EAEA1.2 and DD26.27 exhibited higher predicted binding affinities (−8.5 and −7.9 kcal/mol, respectively) than acarbose (−7.1 kcal/mol). However, this trend was not consistent with the in vitro α-glucosidase inhibition assay, where acarbose demonstrated stronger inhibitory activity (IC_50_ = 0.129 mg/mL) than EAEA1.2 (0.355 mg/mL) and DD26.27 (0.41090 mg/mL). Structural analysis (Figure 9D) revealed that EAEA1.2 did not occupy the same catalytic active site as DD26.27 and acarbose. Instead, EAEA1.2 appeared to bind at a distinct pocket, likely corresponding to an allosteric site. Binding at an allosteric site does not directly compete with substrate binding at the catalytic site but may induce conformational changes that modulate enzyme activity. Therefore, a high docking binding affinity at an allosteric pocket does not necessarily translate into stronger competitive inhibition in enzymatic assays.

The relatively strong predicted binding affinity of EAEA1.2 may be attributed to stabilizing interactions within the allosteric pocket, including hydrogen bonding, van der Waals forces, and extensive hydrophobic contacts. As illustrated in Figure 9C, EAEA1.2 possesses more pronounced hydrophobic regions compared to DD26.27 and acarbose. The hydrophobic environment of the binding pocket may enhance enthalpic stabilization through dispersion forces, while also contributing favorable entropic effects associated with desolvation. These combined interactions could stabilize the enzyme–ligand complex without directly blocking the catalytic site, thereby explaining the discrepancy between docking scores and in vitro inhibitory potency.

#### 2.3.3. Docking of Compounds with β-Glucosidase

**Figure 10:**
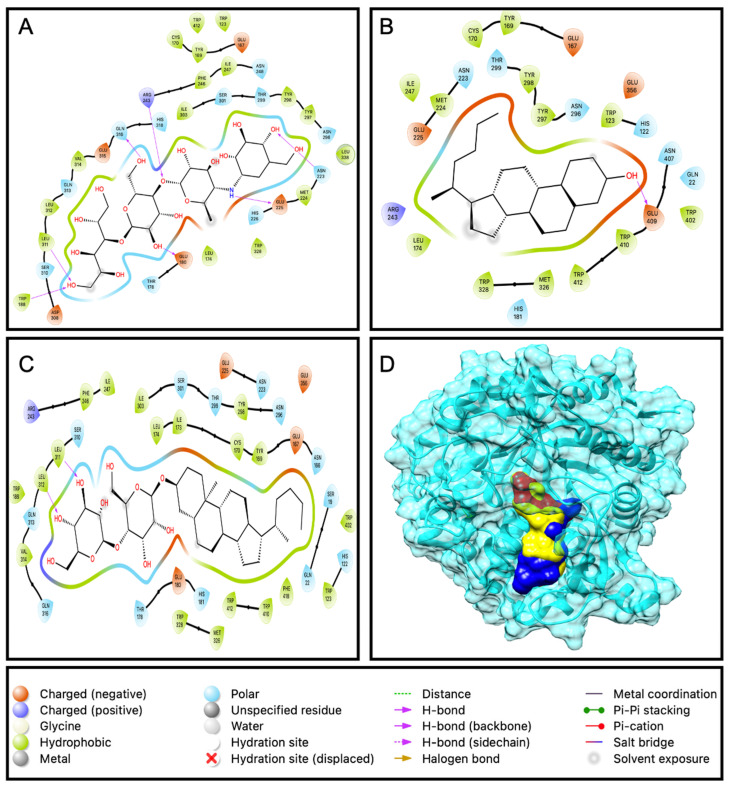
Ligand interaction with the amino acid residues of the active site for (**A**): acarbose, (**B**): DD36.37 and (**C**): EAEA1.2. Box (**D**) represents the protein β-glucosidase and all the ligands bound at the same pocket of the active binding site.

Docking analysis for β-glucosidase indicates that compound EAEA1.2 (−10 kcal/mol) has a higher binding affinity, followed by DD26.27 (−9.2 kcal/mol) and then acarbose with a binding affinity of −7.8 kcal/mol. The results corroborate the findings from the β-glucosidase inhibitory assay results, which indicated stronger inhibition of the enzyme by compound EAEA1.2, with an IC_50_ of 0.34 mg/mL, followed by DD26.27 (0.419 mg/mL) and then acarbose (0.464 mg/mL). The higher binding affinity of the compound EAEA1.2 may be attributed to the high number of hydrophobic amino acid residues they possess, as can be observed in Figure 10C, indicated by green shading. In addition, compound EAEA1.2 binds to many more polar amino acid residues at the active site than DD26.27 and acarbose. As a result, the complex is formed with dipole–dipole and/or electrostatic forces, enhancing the stability and thus improving the binding affinity of the compound. It can also be observed that the standard acarbose established more hydrogen bonding with GLU180, GLU225, ASN223, ARG243, GLN316, LEU311 and TRP198 than the compound DD2627, which established only one hydrogen bond with GLU409, and EAEA1.2, which does not show any hydrogen bonding.

### 2.4. Cytotoxicity of DD26.27 and EAEA1.2 Against Vero and HEK-293 Cell Lines

The cytotoxic effect of isolated compounds DD26.27 and EAEA1.2 was evaluated by MTT. The principle of MTT relies on the ability of mitochondrial dehydrogenase in healthy living cells to convert MTT from normal yellow tetrazolium salt into insoluble purple formazan [20]. The cytotoxic effect was classified on the bases of the proportion of viable cells as follows: if viable cells are >80% = non-cytotoxic, 60% to 80% = weakly cytotoxic, 40% to 60% = moderately cytotoxic and <40% = highly cytotoxic [21,22]. The Vero cells, derived from the kidney epithelial cells of the African green monkey (*Cercopithecus aethiops*) and HEK-293 cell lines derived from human embryonic cells, were used to determine the cytotoxic effects of *M. balsamina*. These cells were used because of their high sensitivity to several toxins and viruses, making them suitable for cell damage studies [23].

#### 2.4.1. Cytotoxicity Against Vero Cell Lines

**Figure 11:**
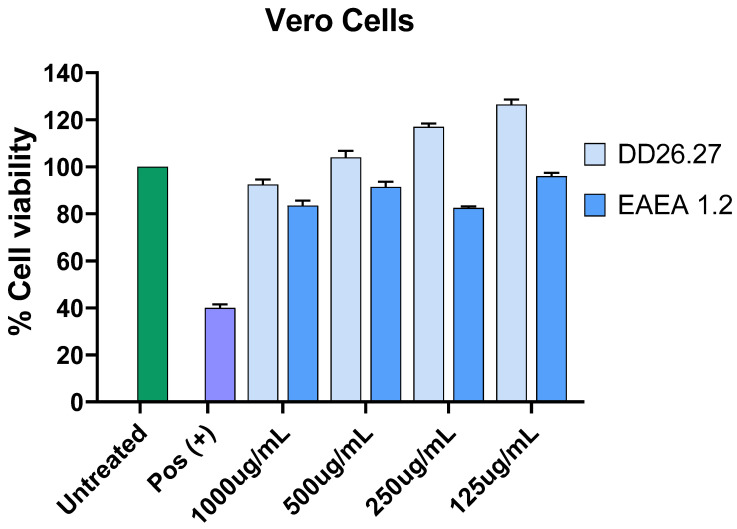
Cell survival following treatment of Vero cells for 48 h with EA L extract, DCM L extract and compounds EAEA1.2 and DD26.27 as determined by MTT assay.

As depicted in Figure 11, the negative control consisting of untreated Vero cells maintained 100% cell viability, confirming normal cell growth and validating the reliability of the assay. In contrast, the positive control, hydrogen peroxide, produced a marked concentration-dependent decrease in relative cell viability. Cell viability dropped below 40% at concentrations ranging from 125 to 500 µg/mL, indicating strong cytotoxicity and confirming the sensitivity of the assay system.

Similarly, the tested compounds DD26.27 and EAEA1.2 demonstrated a mild concentration-dependent reduction in cell viability; however, at all concentrations evaluated, both compounds maintained viability levels above 80%. This suggests that neither DD26.27 nor EAEA1.2 is toxic to Vero cell lines under the tested conditions. Notably, DD26.27 consistently exhibited the highest percentage of cell viability across all concentrations, indicating that it may be the safer compound of the two.

Based on the observed viability values, the IC_50_ (the concentration required to reduce cell viability by 50%) could not be reached within the tested concentration range for either DD26.27 or EAEA1.2, as cell viability remained well above 50%. Therefore, the IC_50_ values for both compounds are estimated to be greater than the highest concentration tested, further supporting their low cytotoxic potential.

Data represent the mean ± standard deviation of two independent experiments. Statistical significance compared to the control group is indicated as * *p* ≤ 0.05, ** *p* ≤ 0.01, *** *p* ≤ 0.001, and **** *p* ≤ 0.0001.

#### 2.4.2. Cytotoxicity Against HEK-293 Cell Lines

**Figure 12:**
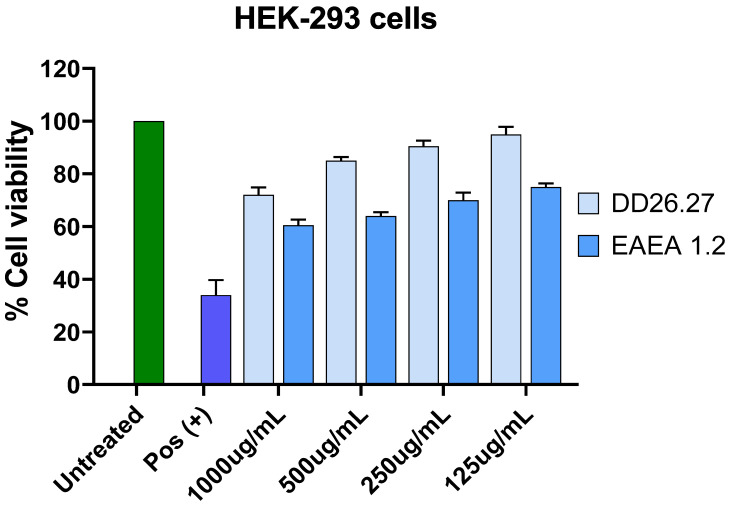
Cell survival following treatment of HEK-293 cells for 48 h with EA L extract, DCM L extract and compounds DD26.27 and EAEA 1.2 as determined with the MTT assay.

Figure 12 illustrates a concentration-dependent reduction in percentage cell viability for the isolated compound DD26.27 and the positive control hydrogen peroxide in HEK-293 cell lines. The negative control, consisting of untreated cells, maintained 100% viability, confirming normal cell growth and validating the assay. In contrast, hydrogen peroxide produced a pronounced decrease in cell viability, dropping from 38% at 125 µg/mL to 26% at 1000 µg/mL, indicating strong cytotoxicity and further confirming the sensitivity of the assay.

Compound DD26.27 demonstrated relatively low toxicity toward HEK-293 cells, maintaining a cell viability above 80% between 125 µg/mL (93%) and 500 µg/mL (84%). However, at the highest concentration tested (1000 µg/mL), viability decreased to 74%, suggesting weak cytotoxicity at elevated doses.

Similarly, compound EAEA exhibited a moderate reduction in cell viability across the tested concentrations. Viability values ranged from 76% at 125 µg/mL to 60–65% at 500 µg/mL, indicating weak toxicity. At 1000 µg/mL, cell viability further declined to approximately 59–62%, suggesting moderate cytotoxicity at the highest concentration.

Based on these results, the IC_50_ value for DD26.27 could not be determined within the tested concentration range, as cell viability remained above 50% even at 1000 µg/mL. Therefore, the IC_50_ for DD26.27 is estimated to be greater than 1000 µg/mL, supporting its relatively low cytotoxic potential. In contrast, compound EAEA approached 50% viability at the highest concentration tested; thus, its IC_50_ is likely slightly above 1000 µg/mL, indicating higher cytotoxicity compared to DD26.27.

Data represent the mean ± standard deviation of two independent experiments, with statistical significance indicated as * *p* ≤ 0.05, ** *p* ≤ 0.01, *** *p* ≤ 0.001, and **** *p* ≤ 0.0001 compared to the control group.

## 3. Materials and Methods

### 3.1. Chemicals and Reagents

All chemicals and solvents used for this study were of analytical grade. The enzymes α-amylase, α-glucosidase, β-glucosidase and standard acarbose were purchased from Sigma-Aldrich, Johannesburg, South Africa. The MTT reagents were also purchased from Sigma-Aldrich SA.

### 3.2. Collection of Plant and Extraction

The leaves of *M. balsamina* were collected from Phake ya Ratlhagane (25°08′51.1″ S 28°30′28.2″ E), situated at Mpumalanga province, South Africa. A sample of the leaves was taken to the South African National Biodiversity Institute (SANBI) for identification. After identification, a voucher specimen MA011 was deposited in the Herbarium of the Pharmaceutical Sciences Unit of the School of Pharmacy, Sefako Makgatho Health Sciences University.

The leaves were air-dried and ground into fine powder (0.91560 kg) that was then serially extracted using the solvents hexane, dichloromethane (DCM), ethyl acetate and methanol starting with the least polar (hexane) to the most polar (methanol) solvent. The extraction process was carried out in 24 h cycles, 2 repeats, on an orbital platform shaker. The obtained extracts were filtered, and we further evaporated the excess solvents with a Stuart rotary evaporator at a temperature of 37 °C and with a speed of 120 cycles per minute. The resultant extracts were left to dry under a stream of air in the laboratory and then stored at room temperature until used.

### 3.3. Fractionation and Isolation from DCM and EA Leaf Extracts

Fractionation and isolation of the compounds were carried out using vacuum liquid chromatography (VLC), column chromatography (CC) and thin-layer chromatography (TLC) to confirm the purity of the compounds. A combination of solvents including hexane, dichloromethane, ethyl acetate, and methanol in different ratios was used to afford enriched extracts. The resulting enriched extracts were then used for the actual isolation via test fraction collections.

#### 3.3.1. Fractionation and Isolation of Compound DD26.27 from DCM Leaf Extract

A total mass of 10.08 g of dry DCM extract was redissolved in 10.0 mL of DCM and subsequently adsorbed onto 14.0 g of dry silica. The mixture was allowed to air dry prior to loading into the column. The column setup included 30 mm od × 2.0 mm wall × 600 mm long B24 sockets and ground glass stoppers B24 with sintered disc P3 and a PTFE S/C glass column (C.C. Immelmann (PTY) Ltd., Robertsham-Gauteng, South Africa). The wet packing method was used to pack the column, whereby 46.0 g of dry silica was mixed with 60 mL of ethanol to form a homogeneous slurry that was filled up to approximately 60% of the total length of a column. This was followed by the addition of the extract–silica mixture, and the column was then connected to a vacuum; next, collection of the eluent to the test tube was assisted with the vacuum pressure for a faster elution. For the DCM column, elution was performed using at least 2 L of solvents of increasing polarities, starting with the least polar solvent pentane, followed by hexane and finally DCM. The collected fractions were concentrated under reduced pressure using a Stuart rotary evaporator (RE400, COLE-PARMER LTD. ST15 OSA, STONE, STAFFORDSHIRE, UK) to remove excess solvent prior to TLC analysis to visualized collected fractions. The mobile phase used for running TLC was hexane: ethyl acetate (8:2 OSA).

#### 3.3.2. Fractionation and Isolation of Compound EAEA1.2 from EA Extract

The same procedure of wet packing, as explained in Section 3.3.1, was used to separate the fractions of ethyl acetate extract. In this case, 10.5 g of ethyl acetate extract was redissolved in ethyl acetate to prepare the extract–silica mixture. Instead of using ethanol to pack the column, hexane was used to prepare the slurry. The fractions were also eluted with solvent of increasing polarity, starting from pentane, hexane, DCM, acetone, ethyl acetate and finally methanol. Collected fractions were monitored with TLC analysis, with chloroform and methanol (9:1 *v*/*v*) as the mobile phase.

### 3.4. High-Pressure Liquid Chromatography (HPLC)

High-pressure liquid chromatography (HPLC) procedure described by Nagarani and coworkers [24] was followed with minor modifications. The isolated compounds were subjected to Shimadzu HPLC-PDA (LC-20AT, Shimadzu Corporation, Kyoto, Japan) used to generate the multivariate absorbance data. This system is equipped with Shimadzu LC-6AD pumps, an SPD-20A prominence UV-vis detector and a LUNA C-118 column (4.6 mm × 250 mm, 5 m). Gradient elution was employed with the mobile phase consisting of solvent A (deionized water with TFA, pH 2.5) and solvent B, which comprised only methanol 99.99%. The gradient used was as follows: 100 to 50% solvent A (0 to 10 min), 50 to 40% solvent A (10 to 20 min) and then 40 to 100% solvent A (20 to 30 min). The flow rate was maintained at 1.0 mL/min, and detection was performed by a UV-diode array detector set at 280 nm. Finally, peak identification was conducted based on the retention time. The operating temperature was kept at 40 °C, and the injection volume was set to 20 μL.

### 3.5. Ultra-Performance Liquid Chromatography–Mass Spectrometry (UPLC-MS)

#### 3.5.1. Preparation of the Sample

The two compounds DD27.27 and EAEA1.2 were soluble in methanol, and thus methanol was used to dissolve them at a ratio of 2:1. The mixture was then vortexed and centrifuged at 4 °C and 14,000 rpm for 15 min. The standards were spiked into the supernatant, which was then directly injected into each of the Waters^®^ Synapt G2 high-definition mass spectrometry (HDMS) LC-MS systems for analysis.

#### 3.5.2. Ultra-Performance Liquid Chromatography–Mass Spectrometry (UPLC-MS)

The two compounds DD27.27 and EAEA1.2 were dissolved in methanol, and thus methanol was used to dissolve them at a ratio of 2:1. The mixture was then vortexed and centrifuged at 4 °C and 14,000 rpm for 15 min. The standards were spiked into the supernatant, which was then directly injected into each of the Waters^®^ Synapt G2 high-definition mass spectrometry (HDMS) LC-MS systems for analysis and the readings were recorded in Table 3 as follows. 

#### 3.5.3. Fourier Transform Infrared Spectroscopy (FTIR)

The Fourier transform infrared spectroscopy (FTIR) spectra were acquired using a Cary 630 FTIR (Agilent Technologies Pty Ltd., Mulgrave, Australia) interfaced with an ATR (attenuated total reflectance) sampling accessory with a single bounce diamond crystal. Spectra, in the absorbance mode, were measured from 4000 cm^−1^ to 600 cm^−1^, by accumulation of 64 scans at a spectral resolution of 4 cm^−1^. A reference (background spectrum of air) was scanned under the same instrumental conditions before each sample measurement. Spectra were processed with Resolution Pro FTIR spectroscopy software (version 5.2.0, Agilent Technologies Pty Ltd., Mulgrave, Australia). Small amounts of the dry compounds were simply placed on the surface of the diamond ATR crystal, and the sample spectrum was collected. For the PCA, the original 1858 spectral intensities were reduced into 254 averaged spectral values, each from five consecutive wavenumbers (dB = 5).

#### 3.5.4. Nuclear Magnetic Resonance (NMR)

The isolated compounds were dissolved in deuterated methanol (CD_3_OD) and transferred to NMR tubes prior to analysis. All spectra were recorded using a Biospin 600 MHz spectrometer using a signal frequency of 150.9 MHz for C-13 and DEPT 135 while all the other experiments used a signal of 600 MHz and were referenced using residual protonated solvent signals (*δ*_H_: 7.260 ppm for CDCl_3_, 3.31 ppm for CD_3_OD and 2.50 ppm for DMSO-*d*_6_; *δ*_C_: 77.000 ppm for CDCl_3_, 49.00 ppm for CD_3_OD and 39.50 ppm for DMSO-*d*_6_). The raw free induction decay (FID) data obtained for the one-dimensional (1H, 13C), distortionless enhancement by polarization transfer (DEPT) and two-dimensional (COSY, HSQC and HMBC) experiments were analyzed and interpreted with the aid of Mestre nova^®^ (Mestre lab Research, S.L. Feliciano Barrera 9B-Bajo, 15706 Santiago de Compostela, Spain) and ACD/Lab’s structure elucidator, Toronto, Canada.

### 3.6. In Vitro Antidiabetic Activity

#### 3.6.1. α-Amylase Inhibition Activity

The α-amylase inhibitory assay was used to assess the effect of *Momordica balsamina* extracts and was determined using a modified version of the standard method described by Sikhakhane and co-workers [25]. Dried extracts were reconstituted in their respective extraction solvents to obtain a stock concentration of 1 mg/mL in a 96-well microplate, and 100 μL of potassium phosphate buffer (pH 7.0) was added to all wells, followed by adding 100 μL of each extract in the first wells of themicroplate, which were then serially diluted across the plate. The positive control, 100 μL of acarbose, was serially diluted using the same method. Subsequently, 20 μL of α-amylase enzyme solution was added to each well, and the plate was incubated at 37 °C for 5 min. After incubation, 50 μL of the substrate 2-Chloro-4-nitrophenyl-α-D-maltotriose was introduced into each well, and the reaction mixture was incubated for an additional 20 min. The absorbance was recorded at 405 nm using a Molecular Devices^®^ microplate reader. The % alpha-amylase inhibition was calculated as follows:

$$\%\;alpha-amylase\;inhibition\;=\;\frac{A_{0}-A_{S}}{A_{0}}\;\times \;100$$

where A_s_ is the absorbance in the presence of the test substance, and A_0_ is the absorbance of the control.

#### 3.6.2. α-Glucosidase Inhibition Activity

A modified version of the Kumar and co-workers [26] method was used to assess the α-glucosidase inhibitory activity of *M. balsamina* extracts and compounds. Then, the re-constituted *M. balsamina* leaf and fruit extracts were serially diluted throughout a 96-well microplate, after 50 μL of potassium phosphate buffer (pH 7.0) was added to each well. Each well was then filled with 100 μL of a commercial glucose test reagent and 50 μL of sucrose solution. An aliquot of 30 μL of intestinal acetone rat powder was added to each well to start the reaction, and the plate was then incubated for 30 min at 37 °C. The same process was used to make acarbose, which was utilized as a positive control. A Molecular Devices^®^ microplate reader was used to measure the absorbance at 505 nm. The percentage inhibition of α-glucosidase activity was calculated as follows:

$$\%\;\alpha -glucosidase\;inhibition=\frac{A_{0}-A_{S}}{A_{0}}\;\times \;100$$

where A_s_ is the absorbance in the presence of the test substance, and A_0_ is the absorbance of the control.

#### 3.6.3. β-Glucosidase Inhibition Assay

The β-glucosidase inhibitory activity of *Momordica balsamina* extracts was assessed at increasing concentrations of 0.2, 0.4, 0.6, 0.8, and 1.0 mg/mL. A total of 120 µL of each concentration was added to a 96-well microplate, followed by the addition of β-glucosidase enzyme. The plate was incubated at 37 °C for 15 min. Subsequently, 20 µL of 2-Naphthyl-β-D-glucopyranoside was added to each well, and the plate was incubated for an additional 15 min at 37 °C. The reaction was terminated by adding 80 µL of sodium carbonate solution to each well. Absorbance was measured at 405 nm using a spectrophotometer. Acarbose, prepared under identical conditions, served as the positive control, and it was tested using the same procedure as the extracts. The percentage inhibition of β-glucosidase activity was calculated as follows:

$$\%\;\beta -glucosidase\;inhibition=\frac{A_{0}-A_{S}}{A_{0}}\;\times \;100$$

where A_s_ is the absorbance in the presence of the test substance, and A_0_ is the absorbance of the control.

### 3.7. Molecular Docking

#### 3.7.1. Retrieval and Preparation of the Ligands

The chemical structures of the isolated compounds were drawn using ChemDraw (Version 20.0; PerkinElmer Informatics: Waltham, MA, USA, 2020) and then exported to Avogadro software, an open-source molecular builder and visualization tool, version 1.XX. This software was used to stabilize the structures by minimizing energy through optimization of the compound geometries. The three-dimensional (3D) structures were uploaded on UCSF Chimera software package V1.14 for further preparations. The preparations included addition of hydrogen atoms, the addition of gasteiger charge and saving the structures as mol2. The standard acarbose was obtained from the PubChem compound database (https://pubchem.ncbi.nlm.nih.gov/, accessed on 2 October 2025) and prepared in the same way as the compounds [27].

#### 3.7.2. Retrieval and Preparation of the Protein

The Research Collaboratory for Structural Bioinformatics (RCSB) Protein Data Bank (https://www.rcsb.org/, accessed on 2 October 2025) was used to retrieve the 3D structures of proteins α-amylase (PDB ID:3BAI; resolution 1.9 Å) and α-glucosidase (PDB ID: 2QMJ; resolution 1.90 Å) in complex with co-crystallized ligands acarbose and β-glucosidase (PDB ID:) in pdb format. The proteins were then uploaded and prepared on UCSF chimera software, and the preparations included removal of all the non-standard residues and unwanted chains, addition of hydrogen atoms and then saving them in protein data bank (pdb) format.

#### 3.7.3. Docking with Auto-Dock Vina

Molecular docking of the prepared ligands and proteins was carried out using Autodock Vina through the UCSF chimera extension [28]. The box size and coordinates were adjusted to include the whole protein to find the most suitable binding pocket. The new grid box dimensions were (center: 5.80181 × 27.4411 × 48.8023; size: 59.0237 × 71.1654 × 60.0836) for α-amylase, (center: −30.793 × 9.10578 × −18.2499; size: 86.0786 × 83.6365 × 82.2404) for α-glucosidase and (center: 65.7215 × 36.0312 × 39.54091; size: 63.1389 × 54.3631 × 63.1933) for β-glucosidase respectively. Several binding poses with different scores were found, and the result with the highest score was selected and saved as a complex. Furthermore, the ligand–receptor complexes were exported to Schrödinger Maestro 2025-3 software, which was used to generate the two-dimensional (2D) ligand–protein interaction, clearly showing the hydrogen and hydrophobic interactions.

### 3.8. Cytotoxicity

#### 3.8.1. Preparation of the Cell Lines

The Vero and Hek-293 cell lines (American Type Culture Collection [ATCC], Manassas, VA, USA) were maintained and cultured in Dulbecco’s Modified Eagle Medium (DMEM) (Sigma, South Africa) supplemented with 10% fetal bovine serum (FBS) and 1% penicillin–streptomycin (PSN). The cells were incubated at 37 °C in a 95% humidified environment containing 5% CO_2_. The culture medium was replaced with fresh medium every 2–3 days until the cells reached 70–80% confluency. Using a light microscope (Nikon TS100, Germany), cell morphology was regularly observed to evaluate cell viability, attachment, mycoplasma contamination, and any morphological alterations. Cell counting was performed using Cell Countess (Thermofisher, USA), at a 1:1 cell to trypan-blue dye ratio.

#### 3.8.2. 3-(4,5-Dimethylthiazol-2-yl)-2,5-diphenyltetrazolium Bromide (MTT) Cytotoxicity Assay

The MTT test was used to evaluate the cytotoxicity of viable cells, after treatment with different concentrations of *M. balsamina* extracts and their isolated compounds in vitro, as described by [29] with minor modifications. The process started by seeding the cell suspension (2.500 cells/mL) into 96-well microplates for 24 h at 37 °C in a humidified environment with 5% CO_2_. This was performed to allow cell adhesion to take place and achieve cell confluency. After 24 h of incubation, a TC20 cell counter (Bio-Rad, Hercules, CA, USA) was used to calculate the number of cells needed for the experiment. Following attachment, cells were treated with different concentrations of DCM and ethyl acetate extracts and their isolated compounds (1, 0.5, 0.25, and 0.125 mg/mL) and further incubated for 24 h under the same conditions. After incubation, the cells were treated with 20 µL of MTT reagent (5 mg/mL in PBS) (Sigma-Aldrich, South Africa), and the plates were incubated for an additional 4 h. The resulting yellow formazan crystals were dissolved in 100 µL of dimethyl sulfoxide (DMSO). The negative control was untreated cells, whereas the positive control was hydrogen peroxide. The absorbance of the formazan product was measured at 560 nm using a GloMax-Multi microplate reader (Promega corporation, Madison, WI, USA). Percentage cell viability was calculated using the formula below:

$$Cell\;viability\;\left(\%\right)=\;\frac{Average\;OD\;(experimental\;group)}{Average\;OD\;(untreated\;group)}\;\times \;100$$

Data for concentration response curves and statistical analysis (One Way-ANOVA) were executed using GraphPad Prism^®^ version 8.4.2, GraphPad Software Inc., San Diego, CA, USA.

## 4. Conclusions

The findings of this preliminary study provide pharmacological evidence supporting the traditional use of *Momordica balsamina* in the management of diabetes. The two compounds, EAEA1.2 and DD26.27, were successfully isolated from DCM and EA leaf extracts and demonstrated notable inhibitory activity against carbohydrate-digesting enzymes, namely α-amylase, α-glucosidase, and β-glucosidase. Molecular docking analysis further substantiated these findings by revealing strong binding affinities and favorable interactions with key active site residues, in some cases exceeding that of acarbose. Additionally, cytotoxicity evaluation provided preliminary safety evidence, as both compounds were non-toxic toward Vero and HEK-293 cell lines at pharmacologically relevant concentrations.

Overall, these results position *M. balsamina*-derived compounds as promising lead candidates for further in vivo validation, including oral glucose tolerance testing in diabetic models, and subsequent preclinical antidiabetic drug development.

## Figures and Tables

**Table 1:** Summarized multiplicity and coupling of integrated DD26.27 protons.

Carbon Skeleton	Carbon-13 NMR of DD26.27	H-1 (Multiplicity)
1	124.4	7.40 (H, d, *J* = 6 Hz)
2	123.9	7.12 (H, d, *J* = 12 Hz, 6 Hz)
3	147.6	Cq
4	119.18	7.58 (H, s)
5	138.5	Cq
6	30.0	2.3, 2.3 (2 H, t, *J* = 12 Hz)
7	26.7	1.36 (2 H, m)
8	31.4	1.33 (H, m)
9	33.54	2.89 (H, m)
10	138.5	Cq
11	30.38	1.33 (2 H, m)
12	27.0	1.31 (2 H, s)
13	33.88	3.21 (H, m)
14	34.4	2.28 (H, dt, *J* = 12 Hz, 12 Hz)
15	29.6	1.27 (2 H, m)
16	29.5	1.27 (2 H, dt, *J* = 18 Hz)
17	32.9	1.33 (H, m)
18	31.24	1.07 (H, m)
19	19.7	0.83 (3 H, m)
20	22.57	1.2 (2 H, m)
21	22.6	1.35 (2 H, m)
22	14.04	0.89 (2 H, m)
23	14.06	0.87 (3 H, m)

**Table 2:** Summarized multiplicity and coupling constant and integrations of EAEA1.2 protons.

Carbon Skeleton	Carbon-13 NMR OF EA.EA	H-1 (Multiplicity)
1	117.2	5.29 (H, d, *J* = 12 Hz)
2	129.4	5.13 (H, dd, *J* = 12 Hz, 11.8 Hz)
3	73.4	3.31 (H, m)
4	30.6	1.87; 1.62 (2H, m)
5	47.9	1.66 (H, m)
6	29.3	2.04; 1.79 (2H, m)
7	114.6	6.67 (H, t, *J* = 12 Hz, 6 Hz)
8	118.0	-
9	48.1	1.97 (H, t, *J* = 8 Hz)
10	49.4	-
11	29.0	1.41; 1.16 (2H, m)
12	24.6	1.38, 1.13 (2H, m)
13	48.4	1.52 (H, m)
14	48.3	2.35 (2, dt, *J* = 4 Hz, *J* = 10 Hz)
15	29.6	1.63; 1.38 (2H, dt, *J* = 4 Hz, *J* = 8 Hz)
16	29.3	1.60; 1.35 (2H, m)
17	67.0	1.28 (H, m)
18	25.7	1.48 (3H, s)
19	30.6	1.31 (H, m)
20	13.3	1.33 (3H, d, *J* = 8 Hz)
21	31.6	2.04; 1.79 (2H, m)
22	129.4	6.39 (H, m)
23	121.5	5.36 (H, m)
24	22.3	1.71 (3H, m)
1′	92.5	4.42 (H, d, *J* = 12 Hz)
2′	76.7	3.73 (H, dd, *J* = 10 Hz, *J* = 4 Hz)
3′	72.4	3.73 (H, dd, *J* = 8 Hz, *J* = 4 Hz)
4′	74.8	3.02 (H, t, *J* = 7 Hz, *J* = 7.2 Hz)
5′	71.5	4.04 (H, m)
6′	61.5	3.79, 3.54 (2H, *J* = 12 Hz)
1″	96.7	5.15 (H, d, *J* = 14 Hz)
2″	73.5	2.14 (H, dd, *J* = 10 Hz, *J* = 4 Hz)
3″	70.5	3.49 (H, dd, *J* = 8 Hz, *J* = 4 Hz)
4″	70.3	3.40 (H, t, *J* = 7 Hz, *J* = 7.2 Hz)
5″	76.5	3.76 (H, m)
6″ 61.5 3.79; 3.54 (2H, *J* = 12 Hz)	61.5	3.79; 3.54 (2H, *J* = 12 Hz)

**Table 3:** The MS, UPLC and gradient system analysis conditions.

MS Conditions		
Detector	Waters Synapt^®^ G2QTOF	
Calibration mass range	50–1200 *m*/*z*	
Capillary voltage	ESI+ 2.8 KV; ESI- 2.4 KV	
Ionization mode	ESI+ and ESI-	
Source temperature	120 °C	
Sampling cone	25 V	
Extraction cone	4.0 V	
Desolvation temperature	350 °C	
Cone gas flow	10.0 L/h	
Desolvation gas flow	600.0 L/h	
Data management	MassLynx™ Version 4.1 UNIFI	
UPLC Conditions		
System	Waters Acquity UPLC^®^	
Column	A Kinetex^®^ 1.7 µm EVO C18 100 Å (2.1 mm ID × 100 mm length)	
Injection volume	5 µL	
Column temperature	50 °C	
Sample temperature	8 °C	
Flow rate	0.3 mL/min	
Mobile phase A	Water + 0.1% formic acid	
Mobile phase B	Acetonitrile + 0.1% formic acid	
Gradient		
Time	%A	%B
	97.0	3.0
	97.0	3.0
	0	100.0
	0	100.0
	97.0	3.0
	97.0	3.0

## Data Availability

All data for this study have been included in this manuscript, and any additional data can be requested from the corresponding authors.
